# Mindful eating and eating behaviours in Greece: exploring the validity and reliability of two mindful eating scales and other eating behaviours for Greek-speaking populations

**DOI:** 10.1007/s40519-023-01615-7

**Published:** 2023-10-21

**Authors:** Michail Mantzios, Konstantinos Zervos, Marsha Koletsi, Kyriaki Giannou

**Affiliations:** 1 College of Psychology, Birmingham City University, C332, The Curzon Building, 4 Cardigan St., Birmingham, B4 7BD UK; 2 Hellenic Center for Mindful Eating-ΕΑΤΤ, Athens, Greece; 3 Department of Psychology, New York College, Athens, Greece; 4 Department of Psychology, De Montfort University, Leicester, UK

**Keywords:** Mindful Eating Behaviour Scale, Mindful Eating Scale, Grazing, Craving, Orthorexia, Emotional eating, Stress eating, Motivations to eat palatable foods

## Abstract

**Background:**

Mindful eating has seen an increase in clinical and non-clinical practices of changing health outcomes. Meanwhile, the restriction of not having validated scales in other languages proposes a barrier to exploring the impact of mindful eating cross-culturally, and specific to the present project, across Greek-speaking populations, limiting the potential of exploring the association with Mediterranean dieting.

**Methods:**

In the present research, volunteers (*n* = 706) completed online the Mindful Eating Behaviour Scale and the Mindful Eating Scale. A forward–backwards translation, leading to face validity, and was assessed for internal consistency (Cronbach’s Alpha) and followed up by an assessment of the factorial structure of the scales. Divergent and convergent validity was explored using motivations to eat palatable foods, grazing, craving, Dusseldorf orthorexia, Salzburg emotional eating, and the Salzburg stress eating scales.

**Results:**

Results indicated that both scales displayed good internal consistency, and the assessment of the factorial structure of the scales was equally good and semi-consistent with the English versions, with parallel analyses and item loadings proposing problems that have been shown in critical review literature. Associations of mindful eating scales to other eating behaviours were replicated to previously established findings with English-speaking populations.

**Conclusions:**

Findings that deviated from the expected outcomes are central to the discussion on the measurement of mindful eating, and further direction highlights the way forward for researchers and clinicians.

**Level V:**

Descriptive studies.

**Supplementary Information:**

The online version contains supplementary material available at 10.1007/s40519-023-01615-7.

## Introduction

The existing psychometric measurements for mindful eating propose substantially different notions of mindful eating. There is still considerable work necessary to objectively measure mindful eating across different populations, but for now, two commonly accepted measures serve cross-cultural inquiries on attentive and non-judgmental eating. Mindful eating emerged from the principles and techniques of secular mindfulness, which according to Kabat-Zinn [1] is the awareness that arises from intentionally paying attention to the present moment without judgment, and observing the unfolding of present-moment experiences. Framson et al. [2] provided the widely used definition of mindful eating as "the non-judgmental awareness of the physical and emotional sensations linked to eating" (p. 1439). The Mindful Eating Scale (MES) [3] was developed with the intention of replacing the Mindful Eating Questionnaire (MEQ) [2] because of rising concerns about the validity. More recently, the Mindful Eating Behaviour Scale (MEBS) [4] was developed in recognition of limitations with the MES such as not reflecting mindfulness theory, despite the MEBS being limiting in other ways, such as investigating decision-making rather than behaviour. Recently, Mantzios [5, 6] highlighted the problems with the MEBS, but also expanded on the lack of utility of other mindful eating psychometric tools (e.g., [7, 8]). The lack of consensus among researchers on definitions and measurements of mindful eating is unfortunate. However, the two psychometric tools mentioned currently offer the most comprehensive and flexible approaches to measuring mindful eating.

The utility of measuring mindful eating significantly expands the knowledge of experimental and longitudinal studies and allows for an objective measurement of performance/success in randomised trials and the verification of mindful eating practices. Experimental research has taken a more detailed approach to explore the individual practices used in mindful eating interventions and programmes (e.g., [9, 10]), such as the raisin exercise and the mindful construal diary/reflection practice (e.g., [11–13]). Participants have overcome obesogenic environments [11], have eaten lower amounts of unhealthy foods [14–16], and have found the joy in eating [17]; all when mindful eating practices were utilised. The ability to enable healthy and moderated eating amid mindless overeating and obesogenic environments may be a way of advancing science, especially when considering the potential of eating mindfully in a setting surrounded by Mediterranean foods and eating traditions [18]. Greece proposes a good example of a nation that follows a Mediterranean diet [19, 20], and offers an opportunity for valuable cross-cultural research across eating behaviours (e.g., [21]).

Research on mindful eating, and more generally, eating behaviour has stagnated in Greece over the past years, mostly due to the unavailability of valid and reliable psychometric tools for quantitative research. The primary aim of this project was to translate and validate two mindful eating questionnaires in Greek (i.e., the Mindful Eating Behaviour Scale and the Mindful Eating Scale). Starting with a forward–backwards translation, leading to face validity, assessment of internal consistency (Cronbach’s Alpha), and finally, an assessment of the factorial structure of the scales, this research aimed to provide valid and reliable measures of mindful eating in Greek, which are consistent with the English versions. A secondary aim became apparent when examining divergent and convergent validity. The availability of other eating behaviour questionnaires validated in the Greek language was limited, forcing a similar forward–backwards translation, as well as an assessment of face validity and internal consistency of a palatable eating motives scale [22], a grazing scale [23], a craving scale [24], an orthorexia scale [25], an emotional eating scale [26], a stress eating scale [27], and an Epicurean eating scale (inclusive of a preference for supersizing sub-scale; see [28]). Based on corresponding research employing English-speaking samples, we expected motivations to eat palatable foods, emotional eating, cravings, grazing, preference to supersize, stress-induced eating and orthorexia to relate negatively to mindful eating measures (e.g., [29–35]). Meanwhile, when the preference for supersizing is seen as the opposite polar of Epicurean eating (see [28]), and Epicurean eating has a description of preference for quality and appreciation of the sensation of food and eating, we expected a positive relationship to mindful eating. Collectively, the findings of this research aimed to provide a baseline for valid and reliable psychometric tools on eating behaviours, and potentially contribute to the expansion and development of knowledge on mindful eating (e.g., [6, 33, 36]); all to be used in quantitative research that relates to mindful eating and/or eating behaviours.

## Methods

### Participants

Seven hundred and six (*n* = 706) participants responded to an online recruitment call for Greek-speaking participants to help with the ‘validation of questionnaires in the Greek language (from English)’ and ‘questionnaires that look into eating behaviours’. Advertisement recipients in social media were asked to promote to others in their social circles, with the intention of creating a snowball effect. Three participants did not complete the questionnaires, and 255 participants were automatically excluded due to currently suffering from a psychological and/or eating disorder. Of the 451 participants, 122 were male, with an overall sample mean age of 35.28 (*SD* = 29.69) and Body Mass Index mean of 23.68 (*SD* = 2.75). The ethnicity of 431 participants was Greek, Cypriot (*n* = 2), Romanian (*n* = 1), Albanian (*n* = 2), and 15 participants did not disclose their ethnic origin. Most participants were omnivores (*n* = 355), while 18 disclosed that they were vegetarians, three were vegans, 10 followed a gluten-free diet, 36 followed other non-listed diets, and 29 did not disclose the diet that they were following. Participants were not compensated for their participation, and the average time taken to complete this study was 21 min (*SD* = 7 min).

### Materials

*Participant information sheet* Participants were asked to report their age, gender, height, weight, ethnicity, and former and current diagnoses of mental health and eating disorders. Participants reporting a younger age below 18, and/or individuals with a current mental illness/eating disorder diagnosis were automatically excluded from the sample.

*Mindful Eating Behaviour Scale* (*MEBS*) [4] The 17-item MEBS measures four domains of eating: Focused Eating (5 items), Eating in response to Hunger and Satiety (5 items), Eating with Awareness (3 items), and Eating without Distraction (4 items), with a sample item being ‘I multi-task when I am eating’ (reverse score item). Answers range from 1 (never) to 5 (very often), and higher scores indicate a higher level of mindful eating. Winkens and colleagues [4] provided detailed analyses on the validity and reliability of the scale, and follow-up analyses verified the initial findings [37], including the validation and factorial structure in the English language (see [38]), and equivalent versions in other languages [39].

*Mindful Eating Scale* (*MES*) [3] The MES is a 28‑item scale, using a Likert scale ranging from 1 (never) to 4 (usually). Sample items are ‘When I feel anxious, I find myself eating’ and ‘I stay aware of my food whilst I’m eating’. Higher scores represent higher mindful eating. Hulbert-Williams and colleagues [3] provided data on the scale during initial validation, and later research indicated that the scale was reliable [34, 40], with initial validations in other languages suggesting equivalency, although with fewer items (see [41]).

*Salzburg Emotional Eating Scale* (*SEES*) [26] This 20-item scale was developed to measure food intake in response to emotional experiences and assesses four emotional states: happiness, sadness, anger and anxiety. Each item, such as ‘When I feel happy…’, is scored from 1 (I eat much less than usual) to 5 (I eat much more than usual). Higher scores indicate that individuals eat more in response to those emotions.

*Epicurean Eating Scale* (*EE*) [28] The Epicurean Eating Scale measures the individual tendency to value epicurean eating pleasures focusing upon the aesthetic appreciation of the sensory and symbolic value of the food. The scale focuses on both epicurean eating tendencies (7 items ‘If I try, I can clearly and easily imagine the taste of many dishes), and Preference For Supersizing (PFS; 6 items, e.g., ‘I often wish I had the option to choose smaller portions in restaurants, reversed item). Answer options ranged from 1 ‘totally disagree’ to 7 ‘totally agree’.

*Salzburg Stress Eating Scale* (*SSES)* [27] The 10-item Salzburg Stress Eating Scale (SSES) assesses eating in response to stress. The scale presents stressful situations, such as ‘On days where everything seems to go wrong,…’, and asks individuals how they would react, with responses ranging from 1 (I eat much less than usual) to 5 (I eat much more than usual). Higher values represent eating more when stressed.

*Grazing Scale* (*GS*) [23] The 8-item Grazing Scale investigates repetitive eating of small amounts of food. A sample item is ‘Have you ever felt compelled or driven to eat, even when not hungry?’, and the responses range from 1 (rarely) to 5 (all of the time).

*Palatable Eating Motives Scale* (*PEMS*) [22] The 19-item PEMS assesses motives for eating palatable but unhealthy foods for reasons outside of hunger; these four motives acknowledge that individuals can consume the listed food and drink due to coping; reward; social; and conformity motives. Sample items include ‘I consume these foods/drinks to forget my worries’ and ‘I consume these foods/drinks to get a “high like” or euphoric feeling’, which are scored on a 5-point Likert scale ranging from 1 (never/almost never) to 5 (always/almost always).

*Food Craving Questionnaire-Trait* (*FCQ-T*) [24] The 15-item FCQ-T is scored on a 5-point Likert scale, ranging from 1 (strongly disagree) to 5 (strongly agree). A sample item is ‘Whenever I have cravings, I find myself making plans to eat.’. Higher scores indicate greater craving.

*The Düsseldorf Orthorexia Scale* (*DOS*) [25] The DOS consists of 10 items, with a sample item being ‘If I eat something I consider unhealthy, I feel really bad’. Items are scored on a 4-point Likert scale, ranging from 1 (this does not apply to me) to 4 (this applies to me). Higher scores indicate greater orthorexia, with a cutoff score ≥ 30 to indicate the presence of ON.

### Procedure

#### Translation procedure

Forward–backwards translation is the most applied process to effectively translate psychometric tools [42]. First, the procedure involved a forward translation from the original language (i.e., English) to the intended language (i.e., Greek). Second, the intended language (Greek) was then translated back into the original language (i.e., English) and compared to the original version. Inaccuracies were identified through differences in meaning that occurred in the backward translation (see also [43]), and potential differences in items were retranslated until full agreement was achieved between the authors and an independent translator who assisted this process. All psychometrics used in this research followed the same procedure to ensure the tools were equivalent to the English versions.

#### Analytical procedure

There were two aims of the present research. First, the internal consistency was tested through Cronbach’s alpha analyses to ensure that all the scales were reliable. Furthermore, the factorial structure was explored to ensure that the Greek population responded in a similar fashion and the factors loaded equally well. Follow-up confirmatory analyses were conducted on both mindful eating scales as factors were described in the original research. In more detail, data screening was conducted prior to inferential analyses to evaluate whether assumptions were met regarding the presence of outliers, multivariate normality, linearity, and homogeneity of variance. The Kaiser–Meyer–Olkin (KMO) measure of sampling adequacy and Bartlett's test of sphericity were also evaluated to ensure data fitness. After all assumptions were met, Exploratory Factor Analysis (EFA), with principal axis factor extraction and oblique rotation was performed. Scree plot identification, Eigenvalues (> 1), and high item loadings (greater than 0.30) were criteria to evaluate factor extraction, with the addition of a Monte Carlo PCA for parallel analysis indicative of rejecting or accepting factors [44, 45]. Once the factor structure was identified, Pearson's correlations between the subscales were performed to investigate the potential of an overall score calculation for the scale, as well as Cronbach's α internal consistency coefficients were calculated for the overall scale (i.e., MES) and subscales (i.e., MES and MEBS). For the MES, subscales were first-order latent factors that loaded onto a second-order latent factor; that is, ‘Mindful Eating Scale’ total. Structural Equation Modelling was run using the maximum-likelihood method, and Confirmatory Factor Analysis (CFA) goodness-of-fit was assessed for this one factor, second-order model, which included the following indices of fit: a Chi-squared by degree of freedom (χ^2^ CMIN/df) ratio < 5; root mean square error of approximation (RMSEA) < 0.08; Adjusted Goodness of Fit Index (AGFI), the Goodness of Fit Index (GFI), Tucker–Lewis Index (TLI), Comparative Fit Index (CFI), and Incremental Fit Index (IFI) > 0.9; Parsimony Normed Fit Index (PNFI) > 0.5 [46–48]. For the MEBS, a similar protocol was used, but without the second-order latent factor, as the original developers suggested in their research a four factor first order model. Second, interrelations and a parallel comparison to previous findings on mindful eating were discussed, further adding to the convergent and divergent validity of the mindful eating scales. All data analyses were conducted using IBM SPSS 28. Data were further analysed for the CFA using AMOS 24.

## Results

To assess the internal consistency of the scales, Cronbach’s alphas were calculated for all scales. For the MEBS, the Cronbach’s alphas were as follows: Focused Eating (*α* = 0.85), Eating in response to Hunger and Satiety (*α* = 0.86), Eating with Awareness (*α* = 0.87), and Eating without Distraction (*α* = 0.73). The present study produced an overall *α* of 0.87 for the total score of the MEBS. For the MES, the five subscales that were indicated by the developers returned the following *α* values: Acceptance (*α* = 0.86), Awareness (*α* = 0.88), Non-Reactivity (*α* = 0.60), Routine (*α* = 0.68), Distractibility (*α* = 0.86), and Unstructured (*α* = 0.64). The present study produced an overall *α* of 0.85 for the total score of the MES. For the rest of the scales, the Cronbach’s alphas were as follows: Grazing Scale (*α* = 0.90), Craving Scale (*α* = 0.95), Motivations to eat palatable foods (*α* = 0.89), Orthorexia Scale (*α* = 0.86), Stress Eating Scale (*α* = 0.92), Epicurean Eating (*α* = 0.84), Supersizing scale (*α* = 0.62). For motivations to eat palatable foods, Cronbach’s alphas were as follows for the subscales: coping motives (*α* = 0.93); reward enhancement motives (*α* = 0.80); social motives (*α* = 0.80); and conformity motives (*α* = 0.60); while for Emotional Eating, and the assessment of four different emotional states, Cronbach’s alphas were as follows: happiness (*α* = 0.90), sadness (*α* = 0.86), anger (*α* = 0.90) and anxiety (*α* = 0.81), with overall Emotional Eating displaying an alpha equal to 0.83. Overall, the internal consistency of the scales was very good across all scales that were translated into Greek, apart from the non-reactive mindful eating, unstructured mindful eating, conformity motives and supersizing subscales.

As a second step in validating the mindful eating psychometric tools in the Greek language, two Exploratory Factor Analyses were performed to explore the similarity of the Greek versions to the equivalent English factorial loadings of the MES and the MEBS. For the MES, the acceptability of the factorial structures was assessed by exploring the Kaiser–Meyer–Olkin (KMO) and the Bartlett’s sphericity test. The Kaiser–Meyer–Olkin measure of sampling adequacy was 0.85, exceeding the recommended value of 0.6, and Bartlett’s Test of Sphericity being significant (*p* < 0.001) further indicated that the assumptions for a factor analysis were met [49–51]. Principal component analysis revealed the presence of eight components with eigenvalues exceeding 1, explaining 24.4%, 11.2%, 8.8%, 5.7%, 5.6%, 4.2%, 4.0% and 3.6% of the variance. An inspection of the screeplot revealed a break after the third component; however, the parallel analysis indicated that all 5 components should be accepted (see Table 1). To aid in the interpretation of these components, Oblimin rotation was performed. The rotated solution revealed the presence of all components showing several strong loadings (> 0.4), but only partially consistent with previous research (see Table 2). The factor solutions explained a total of 56% of the variance, with acting with awareness, awareness, non-reactive/routine, acceptance and non-reactive subscales indicative of the strength of the scale, but not with all items as suggested in the original scale loading on the original factor structures. Item 16 originally in the non-reactive subscale, and items 17 and 18 originally in the routine subscale, loaded together onto a new factor. All items on unstructured eating, which were part of an original subscale (items 25–28) were all rejected, and some items as indicated above loaded on other factors. The interpretation of the components is consistent with previous research, but some items did not load as specified in the original and follow-up literature; elements that may relate to the content validity and structure of the scale, which will be discussed in more detail in the discussion. The CFA revealed that the 28 items and the factorial structure proposed for the MES were not a good fit for the proposed model: CMIN/df = 3.63; RMSEA = 0.086; AGFI = 0.73, GFI = 0.78, TLI = 0.79, CFI = 0.79, IFI = 0.79; PNFI = 0.67.

**Table 1:** Comparison of eigenvalues from principal components analysis (PCA) and the corresponding criterion values obtained from parallel analysis for Mindful Eating Scale

Component number	Eigenvalue from PCA	Parallel analysis value	Decision
1	7.087	1.501	Accept
2	3.258	1.431	Accept
3	2.558	1.377	Accept
4	1.645	1.332	Accept
5	1.620	1.292	Accept
6	1.226	1.254	Reject
7	1.147	1.219	Reject
8	1.054	1.118	Reject

**Table 2:** Factor loadings for exploratory analysis with oblique rotation of Mindful Eating Scale and CFA model

	1	2	3	4	5	6	7	8	CFA
Item 1				− 0.788					0.714
Item 2				− 0.840					0.808
Item 3				− 0.590					0.409
Item 4				− 0.825					0.787
Item 5				− 0.747					0.668
Item 6				− 0.744					0.711
Item 7		− 0.788							0.743
Item 8		− 0.770							0.745
Item 9		− 0.882							0.840
Item 10		− 0.886							0.852
Item 11		− 0.719							0.689
Item 12					0.646				0.627
Item 13					0.542				0.393
Item 14					0.796				0.788
Item 15					0.785				0.719
Item 16			0.817						0.062
Item 17			0.841						0.963
Item 18			0.865						0.782
Item 19							0.800		0.417
Item 20							0.822		0.141
Item 21	.634								0.530
Item 22	.851								0.726
Item 23	.885								0.902
Item 24	.864								0.840
Item 25						0.798			0.467
Item 26								0.694	0.536
Item 27								0.836	0.597
Item 28						0.822			0.445

For the MEBS, the acceptability of the factorial structures was assessed by exploring the Kaiser–Meyer–Olkin (KMO) and the Bartlett’s sphericity test. The Kaiser–Meyer–Olkin measure of sampling adequacy was 0.86, and Bartlett’s Test of Sphericity (*p* < 0.001) indicated that the assumptions for a factor analysis were met. Principal component analysis revealed the presence of four components with eigenvalues exceeding 1, explaining 34.3%, 12.8%, 11.2%, and 6.7% of the variance. An inspection of the screeplot revealed a break after the third component, highlighting the multiple items that loaded on eating with awareness, hunger and satiety cues, and focused eating subscales, respectively, to the percentages reported above. Parallel analysis indicated that 3 components should be accepted (see Table 3). Oblimin rotation was performed assuming that there would be an overall correlation between subscales as all of them were claimed to be measuring mindful eating. The analysis indicated strong loadings (> 0.4), but the loading did not fully reflect the subscales indicative in the original Dutch version, and other validation studies proposing similar factorial structures in English (e.g., [38], Table 4). The three-factor solution explained a total of 58.3% of the variance, where eating with awareness, hunger and satiety cues, and focused eating subscales reflected the factorial structure of the original scale, but the eating without distraction subscale despite the high loadings did not uphold the testing against the parallel analysis. The findings not corresponding to the original scales are further deliberated in the discussion. For both the MES and the MEBS, intercorrelations between subscales were significant with small to medium (*r*_range_ = 0.111–0.488) and medium strengths (*r*_range_ = 0.227–0.489), correspondingly, and with the distractibility subscale being problematic to the ME scale. The CFA revealed that the 17-item scale was a good fit for the proposed MEBS model: CMIN/df = 2.39; RMSEA = 0.059; AGFI = 0.90, GFI = 0.93, TLI = 0.94, CFI = 0.95, IFI = 0.95; PNFI = 0.76.

**Table 3:** Comparison of eigenvalues from principal components analysis (PCA) and the corresponding criterion values obtained from parallel analysis for Mindful Eating Behavior Scale

Component Number	Eigenvalue from PCA	Parallel analysis value	Decision
1	5.823	1.351	Accept
2	2.180	1.279	Accept
3	1.910	1.224	Accept
4	1.137	1.179	Reject

**Table 4:** Factor loadings for exploratory analysis with oblique rotation of Mindful Eating Behavior Scale and CFA model

	1	2	3	4	CFA
Item 1			− 0.828		0.698
Item 2			− 0.675		0.722
Item 3			− 0.816		0.722
Item 4			− 0.853		0.738
Item 5			− 0.632		0.739
Item 6		− 0.822			0.845
Item 7		− 0.788			0.774
Item 8		− 0.819			0.794
Item 9		− 0.761			0.611
Item 10		− 0.762			0.732
Item 11	.891				0.923
Item 12	.868				0.639
Item 13	.775				0.835
Item 14				0.718	0.852
Item 15				0.769	0.846
Item 16				0.734	0.518
Item 17				0.614	0.281

Finally, the data were further explored to examine the potential intercorrelations, and the potential convergent and divergent validity of the mindful psychometric tools. Both MES and MEBS correlated similar to the rest of the eating variables. Both related significantly and negatively to grazing, emotional eating, stress eating, motives to eat palatable foods, craving and supersizing. Findings support previous findings with the English equivalent mindful eating scales. A significant difference was the significant positive relationship of the MEBS to Epicurean eating, while the MES did not relate Epicurean Eating (see Table 5).

**Table 5:** Bivariate correlations between mindful eating and mindful eating behaviour with other eating variables

		1	2	3	4	5	6	7	8	9	10
1. BMI		1									
2. MEBS		− 0.203^**^	1								
3. MES		− 0.201^**^	0.200^**^	1							
4. GS		0.270^**^	− 0.576^**^	− 0.511^**^	1						
5. SEES		0.180^**^	− 0.373^**^	− 0.212^**^	0.435^**^	1					
6. SSES		0.252^**^	− 0.352^**^	− 0.191^**^	0.410^**^	0.781^**^	1				
7. FCQ-T		0.316^**^	− 0.499^**^	− 0.496^**^	0.695^**^	0.472^**^	0.444^**^	1			
8. DOS		− 0.152^**^	0.019	− 0.043	− 0.087	− 0.072	0.038	0.167^**^	1		
9. PEMS		0.267^**^	− 0.309^**^	− 0.306^**^	0.466^**^	0.342^**^	0.303^**^	0.517^**^	− 0.120^*^	1	
10. EE		0.027	0.330^**^	− 0.039	− 0.169^**^	− 0.093	− 0.093	0.044	0.070	− 0.014	1
11. PFS		0.197^**^	− 0.197^**^	− 0.136^**^	0.328^**^	0.210^**^	0.183^**^	0.357^**^	− 0.064	0.364^**^	− 0.184^**^

Body Mass Index = BMI; Mindful Eating Behaviour Scale = MEBS; Mindful Eating Scale = MES; Salzburg Emotional Eating Scale = SEES; Epicurean Eating Scale = EE; Preference for Supersizing =  PFS; Salzburg Stress Eating Scale = SSES; Grazing Scale = GS; Palatable Eating Motives Scale = PEMS; Food Craving Questionnaire – Trait = FCQ-T; Düsseldorf Orthorexia Scale = DOS

^**^Correlation is significant at the 0.01 level; ^*^Correlation is significant at the 0.05 level

## Discussion

The present research had two main aims. First, this research aimed to provide valid and reliable measures of mindful eating in Greek that are consistent with the English versions. In detail, the aims were to translate and validate two mindful eating questionnaires in Greek (i.e., the Mindful Eating Behaviour Scale and the Mindful Eating Scale). First, the process started with a forward–backwards translation, leading to face validity, an assessment of internal consistency (Cronbach’s alpha), and finally, an assessment of the factorial structure of the scales. Second, a similar evaluation of eating behaviour questionnaires was conducted through a forward–backwards translation, as well as an assessment of face validity and internal consistency of the motivations to eat palatable foods scale, the grazing scale, the Salzburg emotional eating scale, and the Salzburg stress eating scale to create comparable research for the assessment of convergent and divergent validity.

Results indicated that both scales displayed good internal consistency (Cronbach’s alpha), and the assessment of the factorial structure of the scales was equally good and consistent with the English versions for the largest part, and more so for the MEBS. The four factors loaded in a similar fashion to the original Dutch scale, but the parallel analysis indicated a lack of power for the eating without distraction scale. It is not surprising, as literature has suggested that there is no consistency in measuring mindful eating behaviour when items are not assessing mindful eating behaviour [5, 6, 36]. For example, ‘I multi-task while I am eating’ (Item 16) does not assess the eating behaviour, but more the decision-making prior to eating, and this could be an element influencing the factorial support for this scale. The follow-up CFA indicated a good model fit when assessed as a four-factor model as indicated by the developers of the scale, but without a higher order explanation through a latent variable of overall measuring mindful eating behaviour. The MES performed in many ways worse, where three factors were rejected through the parallel analysis, and the factors that remained indicated that the items loaded on different factors than the ones that were prescribed by the developers of the scale. For example, ‘I need to eat like clockwork’ (Item 16) was an item that loaded originally on the non-reactive subscale, but in the present data set, it loaded with ‘I have a routine for what I eat’ (Item 17) and ‘I have a routine for when I eat’ (Item 18), both of which were leading on the routine subscale. Loading together, these items suggest a shared factor; however, previous research has not indicated such issues. In addition, the unstructured eating factor did not pass the stress test of parallel analysis, and the same was true for the two items (Items 19–20) that loaded together forming a separate factor. These items were part of the original routine subscale, suggesting that there were two types of routine, one potentially more indicative of disordered eating. These results may be closely linked to the fact that only routine and unstructured eating did not produce adequate alpha values when assessing the internal consistency of the scales and subscales. In addition, the CFA indicated that the one-factor second-order model was not an adequate fit as suggested by the developers of the scale. Convergent and discriminant validity did significantly replicate previous findings. The scales used for convergent and discriminant validity matched similar non-clinical research with English-speaking samples, and rationales on potential associations to mindful eating can be found in the literature, such as mindful eating and: motivations to eat palatable foods (e.g., [33, 40]), orthorexia (e.g., [32, 35, 52, 53]), emotional eating (e.g., [30, 31, 52, 54]), cravings (e.g., [29]), and grazing (e.g., [34]). Meanwhile, Epicurean eating, preference to supersize and stress-induced eating appear relevant to mindful eating and implications can be drawn from other literature on mindfulness and different levels of sensory attentiveness (e.g., [28, 55–57]). The results being so significantly similar further indicated that there could be further cross-cultural research with Greek-speaking populations to advance the scientific enquiry around mindful eating.

The lack of face validity of already existing mindful eating scales may be problematic. The inability to separate mindful eating behaviour (i.e., attentive and non-judgmental eating) from other factors such as distractibility put forward a covariance that does not reflect mindful eating [5, 6]. Hunger and satiety, distracted, routine and unstructured eating simply blur the lines as to what exactly we are measuring, and allow for potential pitfalls drawing associations and observing intervention outcomes (see also [7, 8]). For now, these scales appear to be the best available measures of mindful eating, but researchers need to be cautious as to what they are reporting, as the MES is measuring mindful eating and potential other unrelated sub-constructs, while the MEBS is not looking into behaviour exclusively, and proposes potential inflation of associations through the use of hunger and satiety and eating without distraction subscales. A recent, more critical review of the literature has proposed that focusing on “mindful eating behaviour” enables clarity in theory, practice, and measurements [5], and a corresponding scale [36] was later introduced that overcomes all of the barriers in the science of measuring mindful eating.

The present research presents three limitations. First, the cross-sectional nature of the research is not allowing for observations on the effectiveness of measuring change when participants are in experimental, longitudinal and intervention studies. Future research should utilise these scales in studies that are testing mindful eating interventions and compare and identify if the scales are successful in identifying positive change. Second, the recruitment method and the opportunistic sampling are not adequate to draw conclusions on the population, and future research should draw a wider-ranging population in demographic characteristics to enable the continuation of mindful eating research. Third, more detailed demographic information gathered in the present study would have given more insight into associations and the standardising of scales, and future research needs to identify more demographic information, such as socioeconomic status and educational level, as such data may implicate significant differences [58]. In addition, the implications for clinical samples are not indicated in the present research and require further research and validation across Greek-speaking populations with problematic eating diagnoses. While the sample included a substantial number of participants who self-reported the presence of a psychological or eating disorder diagnosis (< 30%), the intention was not to explore such a population, and the lack of follow-up questions did not allow for any further analyses.

The present research provides an opportunity to advance research in eating and mindful eating with Greek-speaking populations and supports the prospects for cross-cultural research. The inability to provide validity that reflects the convergent and discriminant nature of the mindful eating scales mandates future iterations of eating with Greek populations, and potentially the development of more theoretically aligned mindful eating scales. For now, the advancement and continuance of scientific inquiries in eating and mindful eating are certainly feasible and hold an exciting future for researchers in the field (Supplementary material).

### Strength and limits

The present research presents limitations when considering the cross-sectional nature of the analysis, the convenience sample, and the lack of in-depth demographic information. Strengths are the large sample size, the number of psychometric eating assessments that have been forward–backwards translated and a first validation that has been performed, as well as the validation of two mindful eating scales that could be used to advance the field.

### What is already known on this subject?

Mindful eating has been studied quantitatively for the past 15 years but with minimal research conducted in Greece. Research in other nations has shown positive effects on weight regulation and health behaviour change.

### What this study adds?

The present research adds validation of two mindful eating scales, and provides a list of other eating psychometric tools that underwent forward–backwards translation and initial validations. The implications of this are summarised on the opportunities to advance research in eating and mindful eating with Greek-speaking populations, and supports the prospects for cross-cultural research.

### Supplementary Information

Below is the link to the electronic supplementary material.

Supplementary file1 (DOCX 37 KB)

## Data Availability

Data will be available on request from the corresponding author.
