# Supplementary material for: Mindful eating and eating behaviours in Greece: exploring the validity and reliability of two mindful eating scales and other eating behaviours for Greek-speaking populations
Source: Eat Weight Disord. 2023 Oct 21;28(1):85. doi: 10.1007/s40519-023-01615-7 (PMC10590293; doi:10.1007/s40519-023-01615-7)
Supplement: Supplementary file 1 — Supplementary file1 (DOCX 37 KB) [file 40519_2023_1615_MOESM1_ESM.docx]

**Appendix:**

**Mindful Eating Behavior Scale – Greek**

**(Please note that all items are displayed below, but the MEBS items 11, 18 and 19 are not part of the final version).**

Χρησιμοποιώντας τις παρακάτω επιλογές (1 έως 5), σημείωσε δίπλα σε κάθε δήλωση της κλίμακας (1 έως 20) την απάντηση που σε εκφράζει περισσότερο.

1 = ποτέ

2 = σπάνια

3 = μερικές φορές

4 = συχνά

5 = πολύ συχνά

**Κλίμακα**

1. Παρατηρώ γεύσεις και υφή όταν τρώω το φαγητό μου

2. Έχω επίγνωση της τροφής που τρώω

3. Παρατηρώ την όψη της τροφής μου

4. Παρατηρώ τις μυρωδιές και τα αρώματα των τροφίμων

5. Είναι εύκολο για μένα να εστιάζω σε αυτό που τρώω

6. Εμπιστεύομαι το σώμα μου να μου πει πότε να φάω

7. Εμπιστεύομαι το σώμα μου να μου πει τι να φάω

8. Εμπιστεύομαι το σώμα μου να μου πει πόσο να φάω

9. Βασίζομαι στην πείνα μου να μου υποδείξει πότε θα φάω

10. Βασίζομαι στην αίσθηση του κορεσμού να μου πει πότε να σταματήσω να τρώω

11. Εμπιστεύομαι το σώμα μου να μου πει πότε να σταματήσω να τρώω

12. Τσιμπολογάω χωρίς να αντιλαμβάνομαι ότι τρώω

13. Τρώω αυτόματα χωρίς να αντιλαμβάνομαι τι τρώω

14. Τρώω κάτι χωρίς πραγματικά να το αντιλαμβάνομαι

15. Οι σκέψεις μου τείνουν να περιπλανιόνται ενώ τρώω

16. Ενώ τρώω, σκέφτομαι τα πράγματα που πρέπει να κάνω

17.  Κάνω και άλλα πράγματα ενώ τρώω

18. Τρώω στο γραφείο ή μπροστά στον υπολογιστή μου

19. Βλέπω τηλεόραση ενώ τρώω

20. Διαβάζω ενώ τρώω

Αποκωδικοποίηση

Από 1 = ‘ποτέ’ έως 5 = ‘πολύ συχνά’

Για τις ερώτησεις 12 έως 20 ισχύει αντίστροφη βαθμολόγηση.

**Mindful Eating Scale – Greek**

Χρησιμοποιώντας τις παρακάτω επιλογές (1 έως 4), σημείωσε δίπλα σε κάθε δήλωση της κλίμακας (1 έως 28) την απάντηση που σε εκφράζει περισσότερο.

1 = ποτέ

2 = μερικές φορές

3 = συχνά

4 = συνήθως

**Κλίμακα**

1. Επικρίνω τον εαυτό μου για τον τρόπο που τρώω
2. Λέω στον εαυτό μου ότι δεν πρέπει να τρώω αυτά που τρώω
3. Εκτιμώ τον τρόπο που διατρέφομαι ως σωστό ή λάθος
4. Εύχομαι να μπορούσα να ελέγξω την διατροφή μου πιο εύκολα
5. Λέω στον εαυτό μου πως δε θα έπρεπε να πεινάς
6. Εύχομαι να μπορούσα να ελέγξω την πείνα μου
7. Παρατηρώ γεύσεις και υφές όταν τρώω το φαγητό μου
8. Έχω επίγνωση της τροφής μου όταν τρώω
9. Παρατηρώ την εικόνα της τροφής μου
10. Παρατηρώ τις μυρωδιές και τα αρώματα της τροφής
11. Είναι εύκολο για μένα να εστιάσω στο τι τρώω
12. Μπορώ να αντέξω την πείνα μου για λίγο
13. Όταν έχω αποφασίσει ότι θα φάω, θα το κάνω αμέσως
14. Όταν πεινάσω δε μπορώ να σκεφτώ τίποτε άλλο
15. Nευριάζω εύκολα αν χρειάζομαι τροφή
16. Θέλω να τρώω σε σταθερές ώρες
17. Έχω ρουτίνα στο τι θα φάω
18. Έχω ρουτίνα στο πότε θα φάω
19. Τρώω το ίδιο πράγμα την ίδια ημέρα της εβδομάδας
20. Κάθε μέρα τρώω το ίδιο μεσημεριανό
21. Τρώω ενδιάμεσα χωρίς να έχω επιγνωση ότι τρώω
22. Δεν παρατηρώ το τι τρώω γιατί ονειροπολώ, ανησυχώ ή με διακόπτουν
23. Τρώω «αυτόματα» χωρίς να έχω επίγνωση του τι τρώω
24. Τρώω κάτι χωρίς πραγματικά να το καταλαβαίνω (αναγνωρίζω)
25. Κάνω και άλλα πράγματα ενώ τρώω
26. Κάνω μικρά γεύματα όταν βαριέμαι
27. Τρώω μεταξύ γευμάτων
28. Τρώω στο γραφείο ή μπροστά στον υπολογιστή

Αποκωδικοποίηση

Από 1 = ‘ποτέ’ έως 4 = ‘συνήθως’

Για τις ερώτησεις 1 έως 6 και 13 έως 28 ισχύει αντίστροφη βαθμολόγηση.

**Salzburg Emotional Eating Scale - Greek**

Χρησιμοποιώντας τις παρακάτω επιλογές (1 έως 5), σημείωσε δίπλα σε κάθε δήλωση της κλίμακας (1 έως 20) την απάντηση που σε εκφράζει περισσότερο.

1 Τρώω πολύ λιγότερο από το συνηθισμένο

2 Τρώω λιγότερο από το συνηθισμένο

3 τρώω όσο συνήθως

4 τρώω περισσότερο από το συνηθισμένο

5 Τρώω πολύ περισσότερο από το συνηθισμένο

**Κλίμακα**

1. Όταν αισθάνομαι αισιόδοξος, ...

2. Όταν είμαι ευτυχισμένος, ...

3. Όταν είμαι χαρούμενος, ...

4. Όταν είμαι περήφανος, ...

5. Όταν αισθάνομαι αυτοπεποίθηση, ...

6. Όταν νιώθω μοναξιά, ...

7. Όταν νιώθω κατάθλιψη, ...

8. Όταν είμαι λυπημένος, ...

9. Όταν βαριέμαι, ...

10. Όταν είμαι απογοητευμένος, ...

11. Όταν είμαι έξαλλος, ...

12. Όταν είμαι θυμωμένος, ...

13. Όταν είμαι ενοχλημένος, ...

14. Όταν είμαι εκνευρισμένος, ...

15. Όταν ζηλεύω...

16. Όταν έχω υπερένταση, ...

17. Όταν είμαι αγχωμένος, ...

18. Όταν ανησυχώ, ...

19. Όταν είμαι νευρικός, …

20. Όταν νιώθω άβολα,...

Αποκωδικοποίηση

Ερωτήσεις 1 έως 5 μετρουν Happiness, 6 έως 10 Sadness, 11 έως 15 Anger, και 16 έως 20 Anxiety. Οι υψηλότερες βαθμολογίες υποδεικνύουν πιο έντονο emotional eating.

**Salzburg Stress Eating Scale – Greek**

Χρησιμοποιώντας τις παρακάτω επιλογές (1 έως 5), σημείωσε δίπλα σε κάθε δήλωση της κλίμακας (1 έως 20) την απάντηση που σε εκφράζει περισσότερο.

1 Τρώω πολύ λιγότερο από το συνηθισμένο

2 Τρώω λιγότερο από το συνηθισμένο

3 Τρώω όσο συνήθως

4 Τρώω περισσότερο από το συνηθισμένο

5 Τρώω πολύ περισσότερο από το συνηθισμένο

**Κλίμακα**

1. Όταν είμαι κατακλυσμένος με πράγματα που πρέπει να κάνω,…

2. Σε περιόδους μεγάλου στρες,…

3. Όταν αισθάνομαι ότι τα πράγματα είναι εκτός ελέγχου,…

4. Στις μέρες όπου όλα φαίνονται να πηγαίνουν στραβά,…

5. Ενώ προετοιμάζομαι για μια κουραστική εργασία,…

6. Όταν είμαι υπό πίεση,…

7. Όταν νιώθω νευρικότητα και άγχος, ...

8. Όταν νιώθω ότι δεν έχω καμία επιρροή στα σημαντικά πράγματα στη ζωή μου,…

9. Όταν νιώθω ότι δεν έχω τα πραγματα υπο έλεγχο,…

10. Όταν νιώθω ότι οι δυσκολίες συσσωρεύονται τόσο πολύ που δεν μπορώ να τις ξεπεράσω,…

Αποκωδικοποίηση

Οι υψηλότερες βαθμολογίες υποδεικνύουν πιο έντονο stress eating.

**Grazing Scale - Greek**

**Eρωτηματολόγιο τσιμπολογήματος-συνεχούς μασουλήματος**

1/ Τσιμπολογάς μεταξύ των γευμάτων (π.χ.: να τρως επανειλημμένα μικρές ποσότητες τροφής)

2/ Τρως λίγο ή πολύ συνεχόμενα κατά την διάρκεια της ημέρας ή κατά ένα μεγάλο χρονικό διάστημα της ημέρας (π.χ.: το απόγευμα)

3/ Συνηθίζεις μετά το πέρας του γεύματος να προσθέτεις και άλλη μερίδα ή να τσιμπάς επιπλέον φαγητό

4/ Θα περιέγραφες τον τρόπο που τρως ως απρογραμμάτιστο και επαναλαμβανόμενο;

5/ Θεωρείς ότι συνεχώς τσιμπολογάς

6/ Ένιωσες ποτέ μια έντονη τάση για φαγητό, χωρίς να πεινάς

7/ Ενιωσες ποτέ ανίκανος στο να σταματήσεις να μασουλάς συνεχώς

8/ Ένιωσες να χάνεις τον έλεγχο ενώ μασουλούσες/τσιμπολογούσες

Αποκωδικοποίηση

Από 0 = ‘ποτέ’ εως 4 = ‘συνεχώς’ (Η ερώτηση 3 δεν συμπεριλαμβάνεται στην κλίμακα).

Οι υψηλότερες βαθμολογίες υποδεικνύουν πιο έντονο grazing.

**Palatable Eating Motives Scale- Greek**

**ΚΛΙΜΑΚΑ ΔΙΑΤΡΟΦΙΚΩΝ ΜΟΤΙΒΩΝ ΓΕΥΣΤΙΚΩΝ ΤΡΟΦΙΜΩΝ**

ΟΔΗΓΙΕΣ (παρακαλώ διαβάστε προσεκτικά):

Στην παρακάτω λίστα αναφέρονται λόγοι για του οποίους οι άνθρωποι καταναλώνουν νόστιμες/ λαχταριστές τροφές ή ποτά όπως:

- Γλυκά, όπως παγωτά, σοκολάτα, doughnuts, μπισκότα, κέικ (τούρτες), ζαχαρωτά, muffins, brownies, **λουκουμάδες, πάστες, κρέπες γλυκές,** και άλλα γλυκίσματα
- Σνακ, όπως πατατάκια, pretzels, κρακεράκια, φιστίκια, σταφίδες, και **πιτάκια σφολιάτας,** φυστικοβούτυρο
- Fast foods ή οποιοδήποτε εύκολο και γρήγορο φαγητό που παρασκευάζεται στο σπίτι όπως: hamburgers, cheeseburgers, πίτσα, τηγανητό κοτόπουλο, τηγανητές πατάτες, **σάντουιτς, γύρος, τοστ, σουβλάκια,** onion rings, snack wraps, τηγανητές φτερούγες, και τηγανητά.
- Ανθρακούχα ποτά, αναψυκτικά που περιέχουν ζάχαρη , ενεργειακά ποτά/αναψυκτικά που περιέχουν καφεΐνη

Αναλογίσου τον τελευταίο χρόνο τις φορές εκείνες που κατανάλωσες κάποιο από αυτά τα γευστικά φαγητά/ποτά και πόσο συχνά θα ανέφερες ότι έφαγες ή ήπιες ΓΙΑ ΤΟΥΣ ΠΑΡΑΚΑΤΩ ΛΟΓΟΥΣ.

Χρησιμοποιώντας τις παρακάτω επιλογές, σημείωσε στις 20 δηλώσεις της κλίμακας εκείνη την απάντηση που σε εκφράζει περισσότερο.

1 = ποτέ/σχεδόν ποτέ

2 = μερικές φορές

3 = κατά το ήμισυ

4 = περισσότερες φορές

5 = σχεδόν πάντα/πάντα

**Κλίμακα**

1/ Καταναλώνω αυτά τα ποτά/φαγητά για να ξεχνώ τις ανησυχίες μου.

2/ Καταναλώνω αυτές τις τροφές/ποτά γιατί η οικογένεια μου ή οι φίλοι μου θέλουν να τα καταναλώσω (τα φαγητά ή να πιώ τα ποτά).

3/ Καταναλώνω αυτά τα φαγητά ή ποτά γιατί με βοηθούν να απολαύσω ένα πάρτυ/εκδήλωση.

4/ Καταναλώνω αυτές τις τροφές/ποτά γιατί με βοηθούν όταν νιώθω θλιμμένος ή νευρικός.

5/ Καταναλώνω αυτές τις τροφές/ποτά για κοινωνικούς λόγους.

6/ Καταναλώνω αυτές τις τροφές/ποτά, για να ευθυμήσω, όταν έχω κακή διάθεση.

7/ Καταναλώνω αυτές τις τροφές/ποτά, για μου αρέσει το πως νιώθω μετά.

8/ Καταναλώνω αυτές τις τροφές/ποτά για να μην με «κοροϊδεύουν»/παρενοχλούν οι άλλοι ότι δεν τα τρώω ή πίνω.

9/ Καταναλώνω αυτές τις τροφές/ποτά, γιατί με ενθουσιάζει.

10/ Καταναλώνω αυτές τις τροφές/ποτά, για να νιώσω ευφορία.

11/ Καταναλώνω αυτές τις τροφές/ποτά γιατί κάνουν τις συνευρέσεις πιο χαρούμενες.

12/ Καταναλώνω αυτές τις τροφές/ποτά, για να είμαι μέλος μιας ομάδας ανθρώπων που προτιμώ.

13/ Καταναλώνω αυτές τις τροφές/ποτά, γιατί μ΄ ευχαριστούν.

14/ Καταναλώνω αυτές τις τροφές/ποτά, γιατί κάνουν τα πάρτυ και τους εορτασμούς καλύτερους.

15/ Καταναλώνω αυτές τις τροφές/ποτά, γιατί μου ανεβάζουν την αυτοπεποίθηση.

16/ Καταναλώνω αυτές τις τροφές/ποτά, για να γιορτάσω μια ξεχωριστή περίσταση με την οικογένεια ή τους φίλους.

17/ Καταναλώνω αυτές τις τροφές/ποτά, για να ξεχνώ τα προβλήματα μου.

18/ Καταναλώνω αυτές τις τροφές/ποτά, γιατί έχει «πλάκα» (διασκεδάζω).

19/ Καταναλώνω αυτές τις τροφές/ποτά, γιατί να είμαι αρεστός/η στους άλλους.

20/ Καταναλώνω αυτές τις τροφές/ποτά, για να μην νιώθω παραγκωνισμένος.

Αποκωδικοποίηση

Από 1 = ‘ποτέ/σχεδόν ποτέ’ έως 5 = σχεδόν πάντα/πάντα’

Οι υψηλότερες βαθμολογίες υποδεικνύουν πιο έντονα μοτίβα συμπεριφοράς.

Μοτίβα συμπεριφοράς:

α. Διαχείριση-αντιμετώπιση (coping): 1,4,6,15,17

β. Aνταμοιβή (reward enhancement):7,9,10,13,18

γ. Κοινωνικό (social): 3,5,1,14,16

δ. Συμμόρφωση (conformity) 2,8,12,19,20

**Epicurean Eating- Greek**

1. Αν προσπαθήσω, μπορώ να φανταστώ ξεκάθαρα και εύκολα την γεύση διαφορετικών φαγητών.

2. Οι  φίλοι μου λένε ότι είμαι ένα άτομο που αγαπά τα φαγητά και ενδιαφέρεται πολύ για διαφορετικούς τύπους τροφίμων.

3.Το μαγείρεμα είναι μια σημαντική μορφή τέχνης, παρόμοια με τη μουσική ή τη ζωγραφική.

4. Μου αρέσει  να συζητάμε τη γεύση του φαγητού με τους φίλους μου.

5.Υπάρχει πολύ ομορφιά στα φαγητά.

6. Μπορώ να βρω εύκολα τις λέξεις για να περιγράψω τη γεύση πολλών φαγητών.

7. Περισσότερο από άλλους ανθρώπους, εκτιμώ την όψη, την μυρωδιά, την γεύση, και την υφή των τροφών στο στόμα μου.

8. Συχνά εύχομαι να είχα τη δυνατότητα να επιλέξω μικρότερες μερίδες σε εστιατόρια.

9. Τα μεγέθη της μερίδας σε οικογενειακά εστιατόρια έχουν αυξηθεί υπερβολικά.

10. Μια κανονική μερίδα φαγητού ποτέ δεν φαίνεται να είναι αρκετή για να με ικανοποιήσει.

11.  Είναι σημαντικό η ποσότητα της τροφής στο πιάτο μου να αντιστοιχεί στην τιμή που πληρώνω.

12. Ακόμα και όταν πεινάω, προτιμώ ένα μικρότερο γεύμα με έντονες γεύσεις από ένα μεγαλύτερο γεύμα με λιγότερο έντονες γεύσεις.

13.  Ένα γεύμα που αποτελείται εξ ολοκλήρου από μικρά πιάτα (μικρές μερίδες) είναι λιγότερο ευχάριστο.

Αποκωδικοποίηση

Από 1 = ‘διαφωνώ απόλυτα’ έως 7 = ‘συμφωνώ απόλυτα’

Οι πρώτες 7 ερωτήσεις μετρουν Epicurean Eating ενώ οι υπόλοιπες 6 ερωτήσεις μετρουν Preference for Supersizing (για τις ερωτήσεις 8, 9 και 12 ισχύει αντίστροφη βαθμολόγηση). Υψηλότερα σκορ υποδεικνύουν πιο υψηλό Epicurean Eating και Preference for Supersizing, αντίστοιχα.

**Dusseldorf Orthorexia Scale- Greek**

1.Η υγιεινή διατροφή είναι πιο σημαντική για μένα από την ικανοποίηση και την απόλαυση του φαγητού.

2. Έχω συγκεκριμένους κανόνες διατροφής που ακολουθώ.

3.  Μπορώ να απολαύσω μόνο τρόφιμα που θεωρούνται υγιεινά.

4. Προσπαθώ να αποφεύγω προσκλήσεις από φίλους για φαγητό αν ξέρω ότι δεν δίνουν σημασία στην υγιεινή διατροφή.

5. Μου αρέσει που προσέχω περισότερο την υγιεινή διατροφή από ότι άλλοι άνθρωποι.

6. Αν φάω κάτι που θεωρώ ανθυγιεινό αισθάνομαι (πραγματικά) άσχημα.

7. Έχω την αίσθηση ότι φίλοι και συναδέλφοι με απομακρύνουν λόγω των αυστηρών μου διατροφικών κανόνων.

8. Οι σκέψεις μου περιστρέφονται συνεχώς γύρω από την υγιεινή διατροφή, και αυτό καθορίζει το πως θα οργανώσω την ημέρα μου.

9. Μου είναι δύσκολο να πάω ενάντια στους προσωπικούς μου διαιτητικούς κανόνες.

10. Αισθάνομαι εκνευρισμένος μετά την κατανάλωση ανθυγιεινών τροφίμων.

11. Nιώθω καθαρή/ός όταν τρέφομαι εξ ολοκλήρου με υγιεινές τροφές.

12. Νιώθω αηδία όταν τρώω μη υγιεινές τροφές.

13. Αισθάνομαι χαρούμενη/ος μετά την κατανάλωση υγιεινών τροφών.

Αποκωδικοποίηση

Από 4 = ‘αυτό ισχύει για μένα’ έως 1 = ‘αυτό δεν ισχύει για μένα’

Οι υψηλότερες βαθμολογίες υποδεικνύουν πιο έντονη ορθορεξική συμπεριφορά.

**Craving Scale- Greek**

1. Όταν λαχταρώ κάτι, ξέρω ότι μόλις αρχίσω δεν θα είμαι σε θέση να σταματήσω να τρώω.

2. Αν τρώω κάτι που λαχταρώ, χάνω τον έλεγχο συχνά και τρώω πάρα πολύ.

3. Όταν λαχταρώ κάποιο φαγητό πάντα με κάνει να σκεφτώ τρόπους για να πάρω αυτό που θέλω να φάω.

4. Νιώθω ότι έχω το φαγητό στο μυαλό μου όλη την ώρα.

5. Ασχολούμαι συνεχώς με το φαγητό.

6. Κάθε φορά που λαχταρώ φαγητό, πιάνω τον εαυτό μου να κάνει σχέδια για να φάει.

7. Λιγουρεύομαι φαγητό όταν αισθάνομαι βαριεστημένος, θυμωμένος, ή λυπημένος.

8. Δεν έχω δύναμη να αντισταθώ στην λαχτάρα για φαγητό.

9. Όταν ξεκινάω να τρώω το βρίσκω δύσκολο να σταματήσω.

10. Δεν μπορώ να σταματήσω να σκέφτομαι το φαγητό όσο σκληρά κι αν προσπαθώ.

11. Αν ενδώσω σε μια επιθυμία για φαγητό, χάνω κάθε έλεγχο.

12. Κάθε φορά που έχω μια επιθυμία για φαγητό, συνεχίζω να σκέφτομαι αυτό το φαγητό μέχρι να καταλήξω να το φάω.

13. Αν λαχτάρω κάτι, αναλώνομαι στην σκέψη του φαγητού.

14. Τα συναισθήματά μου με κάνουν συχνά να θέλω να φάω.

15. Είναι δύσκολο να αντισταθώ στον πειρασμό να φάω δελεαστικά φαγητά που είναι γύρω μου.

Αποκωδικοποίηση

Από 1= ‘διαφωνώ απόλυτα’ έως 5 = ‘συμφωνώ απόλυτα’

Οι υψηλότερες βαθμολογίες υποδεικνύουν πιο έντονο craving.
